# Valedictory to the Graduates of Rush Medical College—Feb. 5th, 1862

**Published:** 1862-02

**Authors:** J. W. Freer

**Affiliations:** Professor of Physiology and Surgical Anatomy


### ORIGINAL COMMUNICATIONS.

### VALEDICTORY

TO THE GRADUATES OF RUSH MEDICAL COLLEGE.—FEB. 5TH, 1862.

By J. W. FREER, M. D.,

*Professor of Physiology and Surgical Anatomy.*

GENTLEMEN GRADUATES:

In entering upon the duties of the profession to which you have this day been admitted, it is worth while to ponder them well.

These duties are of a fourfold character—to yourselves as members of a liberal profession, to your patients, to your colleagues, and to the public.

It is trite but necessary to say, that your education has really but just begun. You have been instructed how to observe, what to observe, and how to learn. Your full education is to be the fruit of a lifetime of industry.

The graduate of medicine who goes forth imbued with a spirit of self-sufficiency, deprives himself of that which to the scientific man is the very means of growth and advancement. He fancies himself already fully armed and equipped for any emergency; therefore, what need hath he to rack his *brain* for the purpose of developing new scientific truths? A man of this description, like an automaton, is confined in his performances within the narrow limits prescribed by the artificer. We sincerely hope that there are none such in our present class of graduates, for, Gentlemen, whatever may be your diligence or attainments,—your future honor or eminence,—the time will never arrive when you can rest on your arms and declare the scientific world conquered. There are no weeping Alexanders in the scientific domain.

We doubt not but that, during your pupilage, you have been diligent and careful students. You have unquestionably improved your time in investigating the various sciences pertaining to our profession; but with all the opportunities you have enjoyed, you have but placed yourselves on the threshhold of the temple of medical science—have seen but the passing panorama, vivid though it may have been, yet like it but a fleeting picture. You have perused the traveler's guide book,—have scanned the maps and charts of that vast domain bounded by the *unknown*, and are simply ready to set forth on a journey of observation, verification and discovery. If observant, you will find in your wanderings whole continents of unexplored regions — regions as trackless as the great Sahara.

Wherever your lot may be cast, whether in country, village, or city, you must not expect at once to enter upon a large practice. Leisure, more or less, you will undoubtedly have, and it is this leisure which, according to the use you put it to, will make or mar your future. This leisure should be improved in continuing your studies, taking care to be thoroughly informed in regard to all improvements as promulgated in the various standard medical periodicals.

You will also find ample food for reflection in conning over the various text books. I would also advise you to keep a common-place book in which to note down, at the time, any thing of value or interest.

Now is the time to practice observations with the microscope, an instrument indispensable to the scientific physician, and which, thanks to the optician, can at the present day be secured at trifling cost. You have seen in the progress of the course its immense range of application. By it you can determine the normal structures, and recognize the abnormal—you can see changes in the fluids and solids. The knowledge it will thus give you is inestimable, and beyond it you can apply it every day to detect the character of the medicines you employ, and thus foil both ignorance and fraud.

Supply yourselves also with the more useful chemical reagents, a spirit lamp, a retort and test tubes, and familiarize yourselves with those analyses and tests which, in connection with microscopic observation, have at this day gone far towards establishing both diagnosis and therapeutics as exact sciences. The strong point of the charlatan and quack is in social chicanery and blaring demonstrations of what he has done and can do; the strength of the educated medical man is in his knowledge, gathered by such assistants as these—interrogating nature and demanding possession of its arcana. There is more real power in the microscope and the test tube than in the whole army of empirics, from the first quack who said: "Ye shall not surely die," to the last and meanest follower of Hahneman, who repeats to his deluded dupes a similar promise.

Be not content with mediocrity, but fix your standard high. It is hard work which yields great results. Here and there a man stumbles upon fortune and fame; but industry and perseverance are the only safe guarantors of success. The truly great men in our profession have only become such by toil and unflinching effort; and it is by these only that they have left their impress on the ages in which they lived and sent their names down to posterity with ever increasing honor.

The status of an intelligent physician is such as to involve great responsibility in his relations with the community in which he resides. He is looked upon as the leader of public opinion in all matters pertaining to health or sanitary regulation, and on all kindred subjects his judgment and advice have great weight and influence, and justly so, for it is taken for granted that on these subjects his studies and observations have fitted him to give enlightened opinions. See to it, gentlemen, that you are fully up to the demands made upon you in these respects.

Suffer me to call your attention to other subjects which, though of minor importance in themselves, yet have great influence on your future welfare. In the first place, I would recommend the cultivation of quiet and gentlemanly manners; make yourselves informed in regard to the usages and customs of refined society, and that can only be attained by seeking such for your association, bearing in mind that even the most degraded and ignorant, instructively respect and admire a well-bred gentlemen. I will beg leave to add, that I do not adopt as worthy patterns, the Beau Brommel or Lord Chesterfield style of gentleman,—those polished icicles, all glare and glitter; but the man who, in respecting himself, has a proper respect for the feelings of others.

We now come to speak of the responsible relations existing between yourselves and your future patients: a responsibility greater than any other existing between man and man. Life is the priceless jewel. "What will not man give for his life?" All things earthly kick the beam when weighed in the balance. We must recollect, gentlemen, that we have to deal with what both human and divine laws regard as sacred; a gift of the Creator, which none can blot out or wantonly injure without incurring the weightiest criminality. But suppose that life be lost through neglect or indifference—ignorance of matters connected with mere practical details—think you we shall stand acquitted either at the bar of conscience or of God? It is indeed a solemn thought, even to those who faithfully cultivate and employ the talents with which they have been endowed, that through their deficiencies of knowledge, or of skill, which, with greater diligence, they might have acquired, some valuable life may have been lost to society and the world.

There is, indeed, no calling, save that of the sacred *ministry*, in which the claims of duty and the responsibilities attending its discharge are so weighty and stringent as in ours; claims which cannot be wholly shrouded in forgetfulness or eluded by sophistry.

We may and must frequently err, for human judgment is at best but fallible. But then, if we improve to the utmost the advantages within our reach, if we consecrate our best energies, to the relief of the sick, we shall have at least the consolation of feeling that human lives have never been lost through indifference, indolence or neglect of ours. And here, as in all other cases, we find that interest coincides with duty, and that those who are wholly devoted to the study and cultivation of their art, and who consecrate their entire energies to their profession, are the very men who attain to the greatest eminence, and who are most amply rewarded with the smiles of fortune.

In the words of an eloquent writer, "You are destined to witness many scenes calculated to touch the deepest and most delicately strung cords of human feeling. Feelings of solemnity and awe naturally associate themselves with scenes of suffering and death. Sobs of agony, and sighs of anguish bursting from broken hearts, fair hopes and confiding affections scattered to the winds by the freezing breath of the destroyer, are among the melancholy associations that extend themselves in sombre perspective along the physician's path."

When pestilence is busy in the work of destruction and the love of gain gives place to the love of life, when all that possess the means of flight hurry from the fatal spot, when victims are swept by hundreds into the graves, is it the paltry consideration of fee, think you, that chains the physician to his post? He moves like a ministering angel where the hot breath of contagion wafts poison on its wings, and calmly and fearlessly perils himself to smooth the pillow of a dying wretch, or to administer balm to those who suffer the double burden of poverty and disease. Although he well knows the danger to be silent, insidious and deadly, there is no cowardly shrinking of his heart, no fear of self, but true to the sublime philosophy which he professes, he is ready to yield his life as a pledge of the sincerity of his faith.

You are bound by every moral and sacred obligation to devote your best faculties and energies to your profession. You are to regard human life in every circumstance, in all conditions and aspects, as sacred. You are to bestow the same assiduous attention upon the poor as the rich; the vicious and depraved as the virtuous and good. You are to remember that guilt equally attaches where life is lost, which you might have saved, as if you had been actively and intentionally instrumental in bringing about the same fatal result. You are to do nothing to lessen the confidence that is reposed in you as physicians, or as men; nothing to bring your morals, or integrity in question. You are to be kind and gentle in all your deportment towards the sick; ever calm and circumspect, sympathizing and cordial; avoiding all eccentricities in manners or opinions; affable without meanness; grave without formality; cheerful without levity; never violating the seal of confidence in relation to professional or family matters which may come to your knowledge. You are not to offend the most delicate sensibilities, either by carelessness of manner or indelicacy of speech. You will cheer the desponding, and comfort the dejected, and sustain the hopes of the despairing. Knowing how conducive the sufferings of disease are to salutary reform, you will endeavor, on every fit occasion, to bring back to virtue, as well as to health, those who are sunk in ignorance, misery and crime. You are not expected to usurp the office of the sacred priesthood, but are expected to be inspired with the purest philanthropy, with a tender sympathy, a devoted charity; for these are the bright perennial fountain whence disinterested labors and sacrifices are to flow. You will by turns speak the language of science and morality; ever cheerful and hopeful in the presence of the sick; never relaxing your attentions because the case has become apparently hopeless, for this is the very time when these attentions will be most needed—remembering that it is equally your duty to prolong life, and mitigate pain and suffering, as to cure disease. Never abandon hope even in cases usually considered hopeless; for, as Hufeland well observes, hope generates ideas, elevates the mind to new views, and new endeavors, and even renders impossibility possible. He who has given up hope has given up reflection, to which apathy and paralysis of the mind follow, and the sick must invariably die, because he who has been called to his assistance is already dead.

Even in the stage of dying the physician should not forsake the sick, for even then he may become a benefactor, and if he cannot save, may at least relieve departing life.

Next, I would advert to the duties you owe to your professional brethren. We have, as most of you are probably aware, a code of Medical Ethics, established by the National Medical Association, which is most excellent, and you will find it profitable to carefully peruse it. But to him who would always do right and be right, I know of no better *rule* than the golden one: "Do unto others as you would they should do unto you." One great and prominent reason of the somewhat low estimation in which the profession is held by some persons, is due mainly to the bickerings, envying, and bitter jealousies of many practitioners towards each other. This should by all means be avoided. A physician who is perpetually engaged in disparaging his professional brother, even though he may fancy a just cause, induces a habit of mind that reacts with ten-fold force upon himself; it sours his temper, warps his judgment, and causes him to view everything as distorted by his own distempered imagination. The true physician is never actuated by such a spirit. On the contrary, he is a man of enlarged and comprehensive views—a cosmopolitan, in the complete sense of the word—overlooking the prejudices of all with whom he may be brought in contact in his profession; rendering efficient aid to Jew or Gentile, Infidel or Christian; at home, if duty calls him there, in the lowest haunts of vice, or the most refined and virtuous society.

Your relations with your professional brethren as consulting physicians or surgeons, is a position of exceeding delicacy. On such occasions, oftener than any other, it is, that injustice is perpetrated against a professional neighbor, and oftentimes the perpetrator executes his nefarious designs so artfully and insidiously—as by signs, manner or gesticulation—that you will be at a loss for a basis on which to found a charge of dishonesty or unfairness.

When people demand a consultation, you may rest assured that they have lost more or less of faith in your skill and ability as practitioners of medicine. Already they or their friends have in their mind's eye one whom they consider as the embodiment of profounder wisdom and greater professional capacity than their regular and faithful medical attendant. And on the other hand, where the attending physician deems it necessary to ask professional advice, people deem it a tacit acknowledgement of superiority of skill and capacity on the part of the gentleman whom you may have selected for your advisor. This being the usual condition of feeling under such circumstances, we sincerely hope that no member of this graduating class will ever seize upon this vantage ground against his professional brother, under any pretext.

On the contrary, a consulting physician, if he be a gentleman, will behave on all such occasions with great modesty and reserve, uniformly addressing his remarks to the attending physician, rather than to the family or patient—considering himself as guest and advisor of his professional confrere, rather than of the family or surrounding persons.

It is the height of injustice towards the attending physician, to answer questions such as are usually propounded by friends and patient in regard to the nature of the disease or the correctness of the previous treatment. To refuse, under such circumstances, is not rudeness; on the other hand, to answer them is the depth of meanness and unfairness. Reserve all your professional opinions for the private and confidential conversation between yourselves. If, by mutual agreement, it is determined to change the course of treatment, in justice to your professional friend, do not change it immediately, if it can be avoided with safety to the patient, for such a course will be deemed as evidence that the consulting physician has disapproved of the former management of the case. By pursuing the above course, while you may not supplant your professional neighbor, you will most assuredly secure his friendship and respect for all time to come, as well as the admiration of those who have witnessed your gentlemanly and modest bearing.

Under this head, I would also add that the undiscerning public quite as frequently withdraw their faith from the intelligent and honorable physician, to repose it in the brawling and ignorant charlatan—as with your acknowledged peer.

Your jealous care for the honor of the noble science which you have espoused, as well as your own self-respect, will forever debar you from professional contact with such excrescences of the *ars medendi.*

In a word, your are to scrupulously avoid, at all times and on all occasions, any and all disreputable professional associations; but grapple to your breasts, with hooks of steel, every upright, intelligent and high-minded physician—for in union there is strength. And alas! it is for want of this union—particularly in our own country—that our noble and beneficent calling has been measurably shorn of its sacredness—has been drabbled and draggled in the dust by every aspiring knave—until (I blush to say it) the public have come to look upon pretension as the measure of qualification, and a gaudy tin sign, with staring hieroglyphics, as equal to a diploma from the most renowned institution of the world! Look to it, then, that you guard sacredly the honor and interests of your acknowledged professional brethren, for in doing thus, you will compel public respect, by convincing them that we are not a band of wranglers, but on the contrary harmoniously engaged in the completer development of principles embracing in their scope the highest temporal interests of the human race.

Next; in regard to your relations and responsibilities with the public at large, as separated from your duties and liabilities toward your patients, and you cannot accomplish the great ends of your mission if you leave these duties unperformed.

Ours is the only science which regards the entire man. The divine regards him in a religious and moral aspect, as a responsible being, endowed with reason, conscience and will, and his mission is confined to the control and direction of these faculties. The legal fraternity look upon a man simply in his social capacity, endowed with certain political and civil rights, and owing to society, of which he forms a component part, the performance of certain duties, and the refraining from certain acts, which might infringe upon the rights and interests of others. He looks upon him, especially, as the possessor of goods and chattels, who is bound to regard as sacred the laws of *meum* and *tuum*, under the pains and penalties of sundry acts, laws and statutes, made and provided to meet all cases aforesaid.

But the medical man takes a still wider range—a more comprehensive survey; he commences by learning the physical structure of man; he marks the evidences of design; investigates the functions of the various parts of this wonderful microcosm; he penetrates far beyond those mechanical and chemical principles and laws, which he finds everywhere exhibited in the human structure; explores the nature of those vital properties, the mysterious attributes of life as displayed in living matter; he even goes beyond and above all this, and studies man as an intellectual being, endowed with rare capacities, thought and reason; subjecting the very laws of the universe, and subtlest agents of nature to the control of his will; he even seeks to know the nature of those ties, and the mode of that union, which connects this human soul with the elements of brute matter; how it depends for its development and its healthy exercise on certain conditions of that noble organ—the very throne of intellect—the brain; how when that organ is the seat of derangement and disease, reason totters, and that intellect, which once shone as a meteor, now lies in splendid ruins—reminding us of a Grecian temple of classic beauty, shattered by an earthquake shock or the more ruthless hand of time. He stops not here. He examines all causes, physical or otherwise, that may serve to warp or disarrange his moral or intellectual faculties; he studies man as a being possessed of the highest attributes, of a sense of accountability to a higher Power, and he finds that his religious nature, as well as conscience itself, are subject to physical agencies and corporeal organization.

Trace the true physician to the bed-side of the sick; observe his ministrations to the poor sufferer whose disease is but the legitimate result of unbridled passion and indulgence; the prophylaxis he prescribes is the return to a virtuous life—the abandonment of habits which are sure to prove fatal sooner or later. It is not transcending the limits of his science or his art when he rises to the source of the malady for which he prescribes, and strikes at the very root of the evil, giving a wise caution and a solemn warning, which fall upon the ear with irresistable force, establishing with incontrovertible proof that our profession embraces in its comprehensive scope, the entire man, physical, intellectual and moral.

To the thinking mind it is a marvel that the medical profession, thus comprehensive in scope, and yet so closely interwoven with both public and private interests, upon which such tremendous responsibilities are imposed and such requirements made, nevertheless does not receive that high place in social estimation which it certainly ought to be awarded—nay, more, that its very respectability is contested by parties, either ignorant or depraved, who would drag it down to their own low level, with scarcely a note of remonstrance from the popular voice.

The greatest injustice our profession receives from the public is in being associated with the chameleon-colored hordes of Quackery, and in their gravely discussing whether it is little better than they, not appreciating that between them and us there is a great gulf fixed, wide as that which separated Dives from Abraham. It is noticeable that, like Dives of olden time, they are ever anxious to assert that they also are worthy of being clasped to Abraham's bosom.

It is needless to repeat the fact, obvious to all familiar with the progress of the sciences, whose elucidation is the glory of our age, that no other class of men has a record on their pages of honor equal to the medical profession. We point to the roll of names linked to immortal fame because of their daily contributions to the knowledge which is lifting this age so high above all the centuries of the past, and challenge the production of a like number from any other class; and, we remember that, as Blackstone said, our "profession hath deserved more than any other for general and comprehensive knowledge," and then wonder why it is that any portion of an intelligent community should permit mere sciolists, pretenders and knaves even the semblance of social equality with it.

Gross injustice is done the profession by violation of the first rule of common sense, viz: that those skilled in any art or science are the ones in whom to confide in all questions pertaining to that art or science. In business affairs every man is forced to regard this; it is a rule even in courts of justice. Why should it not be in the great court of public opinion?

But we sometimes hear, "who shall decide when doctors differ?" The reply is obvious, if the disagreement be between true physicians, recognized as such by the great body of educated medical men, it can only be decided as all mooted points in other arts and sciences are decided, by full inquiry, discussion and investigation by experts, certainly not by those totally ignorant of the matter.

What would be thought if honorable judges, differing on some occult point of *law*, should call in the door-keeper of the court to decide; or, if Doctors of Divinity should ask the Hottentot Pagan to solve their knotty arguments?

This, at least, is common sense. The man of education and culture, and the class to which he belongs should receive the confidence and respect of the public, to the total exclusion of that man and that class wholly devoid of these obvious qualifications.

So far as our profession and the various phases of Quackery —Hydropathy, Thomsonism, Homœopathy, &c., &c.,—are concerned, there can, in a strictly scientific point of view, be no room for question; it stands upon the strong foundation of true science.

A singular misapprehension exists in a portion of the public mind in regard to the true character of medicine as a science. Some suppose it to be a system of guesswork, or at best a more or less shrewd and adroit adaptation of means to ends without the guidance of fixed and established principles; that there may be varying systems, better or worse as the case may be, and it is of little import which is adopted, but this is a gross logical error. Science is classified knowledge, facts reduced to series and their relations made known, and there can be but one which is real and true; there can no more be two sciences of medicine than two sciences of logic, of chemistry, or of natural history. Science is kingly and brooks no rival near its throne.

The science of medicine, Gentlemen, as we, your temporary instructors, have attempted to impress upon you, is a catholic system; it embraces all the facts of man's structure, organization, the phenomena of life and all, whether material or immaterial, which influences it in health or disease. The true physician is lord of this whole manor, and it is his province to control all influences to the preservation and prolongation of life. He it is who has discovered the truths which have so wonderfully increased the duration of human life during the historical period. He it is who rests solely on scientific truths, discovered by precisely the same methods employed in other sciences, and applied under precisely the same processes of reasoning which guide all arts founded upon science. Our science is not exclusive, save as truth must ever exclude error; it is not restrictive, save as fact must ever prevent the incursion of falsehood. Its principles, like other great truths of nature, "cannot be caged in the narrow confines of a rhetorical sentence."

The term Allopathic, as characterizing our profession, should be repelled. It is but a nickname, and no scientific medical man can accept it. He should accept of no other appellation save the time-honored and expressive term *Physician.*

Let medical delusionists claim that their schemes are exhaustive, that they have discovered the fundamental laws of disease, and consequently specific, that is unmistakable and infallible methods of cure. The humble scholar whilst, like Newton, compelling the admiration of the world at the grandeur of the truths he has developed, still feels that he has but collected here and there pebbles from the shore of the great ocean of knowledge.

A similar delusion in former times, with similar beauty of simplicity, referred all forms of matter to four elements—earth, air, fire, and water.

All things in this sublunary sphere are subject to disturbances of the great law of order; all living things are subject to disease, from the tiniest moss to the gigantic oak; from the monad to the elephant, and all the living creations of the human intellect in like manner are now and then infested by ignoble vices, begotten by the demon of disorder. Religion itself has its Mormonism and Free-love; politics has its anarchists and its rebels; law has its burglars, its thieves and its murderers. Why should medicine expect an isolated fate, and thus escape its hydropathists, its steam doctors, its Indian doctors, its mesmerists and homœopaths?

It would be just as good sense to cut down the plum tree because it is infested with the curculio, as to ignore rational medicine because empirics fatten upon the result of the stings they inflict. Do not cut down the tree, but destroy the vermin with caustic, or shake them off and trample them in the dust.

Follies and absurdities seem wonderfully out of place in such close juxtaposition with true knowledge, but thus it has been ever since man first began to learn. The vermin never know their place, and will fasten upon the noblest of things; a fact which did not escape the acute perception of the Scottish poet, when attracted by the incongruous position of the "crowlin ferlie" on the lady's bonnet in church, and we are tempt to exclaim with him:

"Ha! whare ye gaun, ye crowlin ferlie!
Your impudence protects you sairly;
I canna say but ye strunt rarely
Owre gauze and lace.
Tho' faith, I fear ye dine but sparely
On sic a place.

"Ye ugly, creepin, blastit wonner,
Detested, shunn'd by saunt and sinner,
How dare ye set your fit upon her,
Sae, fine a lady?
Gae somewhere else, and seek your dinner
On some poor body.

"My sooth! right bauld ye set your nose out,
As plump and gray as onie grozet;
O for some rank mercurial rozet,
Or fell red smeddum,
I'd gie you sic a hearty doze o't
Wad dress your droddum."

The quack, whatever patronymic he baptizes himself with, and almost always his adherent, can be told at a glance; he wears it in his face, his gestures and his attitudes, and if by assiduous caution he deceive in these, "his speech ever bewrayeth him." His are not catholic views. Knave and dupe are upper and under sides of the same coin, with base metal always between. Even if he believes in his own imposture, his credulity will always have the element of knavery in it; or, if he believes it not, his knavery will always wear the motley—the cap and the bells.

If you really know your profession, you must respect it, love it, and seek to honor it. Hence you must sustain its true character and dignity before the public. You cannot compromise the matter—you cannot shirk the responsibility—you cannot give place to the evil, but must rebuke it. It is your duty to enlighten the community in which you live. You have no right to allow them to be duped by designing knaves, or fall the easy victims of low delusion. The community have a right to this service from you, and you are false to the profession, to yourselves, and to your fellow-men, if, seeing the evil, you do not speak the timely word of caution, or the sharper one of denunciation.

You should no more, as honorable men, pander to the vices of the human mind, than to the vices of the human body. The evil is a grave one, and, like Sabbath breaking, profanity, gambling, and some nameless crimes of a darker hue, the moral sense of the community seems to have become obtuse to it by the very multitude of its exhibitions.

The charge is frequently made that medicine, as a science, has been undergoing constant mutations. If by this, it is intended that it is a progressive science, we admit it freely; it is the glory of the profession. But, if by this, it is intended that it has been a history of contradictions—we repel it as begotten either of calumny or ignorance. Methods of accomplishing results have varied and will continue to vary, as men and times, seasons and surroundings change.

The objects sought by Hippocrates, we still seek to-day, and will continue to seek during all coming time. Powers and Turner may and probably do adopt different methods of giving shape to their glowing ideals, from those adopted by Praxitteles and Apelles; but the same eternal principles vitalize all their creations. You may rest assured that were Hippocrates now living, he would be the disciple of no pathy or—ism—even to secure admission to the luxurious apartments of the hysterical or dyspeptic votaries of infinitesimal tittillations.

Gentlemen: allow me to allude to the record of our Alma Mater. It is now nineteen years since our venerated and distinguished colleague and President of this Institution, conceived the plan of a medical college in the city of Chicago. He received but little of encouragement—the project being considered impracticable, if not chimerical; but with characteristic energy and perseverence, he pushed boldly forward, overriding all obstacles, until, from a graduating class of *one*, the number has gradually arisen to forty annually.

The nicest sense of propriety cannot be disturbed by a single allusion to another member of that first Faculty of the College, who has from that time shared all of its fortunes, and to whom it is largely indebted for the high position it has achieved. Whilst some were coldly doubtful in their support, and whilst other walks of professional life have attracted others, and whilst others still have sought to divide, distract, and destroy—he has ever lent his best energies, his unequaled social qualities, and his splendid attainments to the welfare and prosperity of this College. I would say more, but his personal presence forbids. Soon he starts for another field of duty, lending his large scientific and professional knowledge to the support of that National Government under whose benign auspices, not only this College, but all other worthy institutions of our common country, have prospered beyond any degree which history records. I but echo the sentiments of this class and my colleagues, when I bid him the fervent God-speed in the work before him, and breathe an earnest prayer for his safe return to us before our next annual session —"when grim-visaged war shall have smoothed its wrinkled front," and the arts of peace again replace the discordant sounds of civil commotion.

The alumni of this Institution already number something over seven hundred. In almost every nook and corner of the great West, you will find her toiling and earnest sons; and we say it with pride and satisfaction, that few have ever turned aside from her precepts. Many are occupying high positions in Medical Colleges, and a large number are rendering yeoman service in their professional capacity in the grand struggle for our national existence.

Allow me to say in conclusion, that you can scarcely estimate the amount of good that each of you may accomplish in the world, if actuated by proper motives; and in order to this, you must bring yourselves to feel your weighty responsibilities.

The Trustees and Faculty feel, in presenting you with these diplomas, that we are linked with your future—be that of an honorable or dishonorable character. While we have, from association, learned to have confidence in your honor and integrity, yet we would beg leave to give the parting warning—to do naught in the future that may bring discredit upon your Alma Mater. The true teacher ever feels a deep and peculiar interest in his pupils, and we, as having been your preceptors in the noble art of healing, will ever be ready to extend toward you the helping hand. Gentlemen, disappoint us not. Fulfill your destiny; carry out your purposes with a brave heart and manly courage.

> "The world is all before you where to choose
> Your place of work, and Providence your guide."

Farewell.

